# Fezolinetant treatment for hot flashes associated with menopause: A plain language summary of the DAYLIGHT study

**DOI:** 10.1177/17455057261432619

**Published:** 2026-04-03

**Authors:** Marla Shapiro, Rossella E. Nappi, Xuegong Wang, Antonia Morga, Katrin Schaudig

**Affiliations:** 1 University of Toronto, Toronto, ON, Canada; 2 University of Pavia, Pavia, Italy; 3 Research Center for Reproductive Medicine and Gynecological Endocrinology – Menopause Unit, Fondazione Policlinico IRCCS S Matteo, Pavia, Italy; 4 Astellas Pharma Global Development, Northbrook, IL, USA; 5 Astellas Pharma Ltd., Surrey, United Kingdom; 6 Hormone Hamburg, Hamburg, Germany

**Keywords:** menopause, lay summary, neurokinin 3 receptor antagonist, fezolinetant, hot flashes, vasomotor symptoms

## Abstract

What is this summary about?

The DAYLIGHT study took place in Europe and Canada between 2021 and 2023. Researchers looked at the effect of fezolinetant treatment on hot flashes/flushes and night sweats (known as vasomotor symptoms) associated with menopause. Hot flashes are the main reason that people seek medical attention for menopause.

To take part in the DAYLIGHT study, participants had to be assigned female at birth, between 40 and 65 years old, experiencing moderate to severe hot flashes associated with menopause, and unsuitable for hormone therapy either for a medical reason or because they did not want to take it. All participants took either a fezolinetant pill or a placebo. The placebo looked like fezolinetant but did not have medicine in it.

During the study, researchers and participants did not know who was taking fezolinetant and who was taking the placebo.

The DAYLIGHT study was the first to look at how well fezolinetant worked compared with placebo for 6 months. Researchers wanted to find out how many hot flashes participants had each day. They also looked at how severe the hot flashes were, sleep quality, quality of life, mental health, work productivity, and side effects.

What were the results?

Compared with the placebo, fezolinetant reduced the number of hot flashes that participants had each day. Participants taking fezolinetant also had greater improvements in sleep, quality of life, depression, and work productivity. Side effects were similar for participants who took fezolinetant or the placebo in this study.

What do the results mean?

The DAYLIGHT study shows that fezolinetant may be a non-hormonal treatment option to reduce hot flashes and improve quality of life for people in menopause. This adds to the evidence that fezolinetant can help people having hot flashes associated with menopause.

**Click here to view the PDF**

